# O06 Consumption of colistin and fosfomycin in a burn intensive care unit

**DOI:** 10.1093/jacamr/dlag102.006

**Published:** 2026-06-26

**Authors:** Nesrine Hagui, Mariem Gargouri

**Affiliations:** Center of Trauma and Major Burns, Fattouma Bourguiba Hospital, Monastir, Tunisia; Center of Trauma and Major Burns, Fattouma Bourguiba Hospital, Monastir, Tunisia

## Abstract

**Background:**

Antimicrobial resistance represents a major challenge in the management of infections, particularly in intensive care units. Colistin and fosfomycin are considered last-resort antibiotics for the treatment of infections caused by MDR bacteria. The aim of this study was to analyse the prescription and consumption patterns of these antibiotics in the burn intensive care unit (BICU).

**Materials and methods:**

This was a retrospective descriptive study conducted in the burn intensive care unit of the Traumatology and Major Burns Center of Ben Arous over a one-year period from January to December 2024. All patients who received treatment with colistin or fosfomycin were included. Antibiotic consumption was expressed as defined daily doses per 1000 patient-days (DDD/1000 PD), calculated according to the WHO 2024 standards.

**Results:**

A total of 77 patients were treated with colistin, with a consumption of 95.88 DDD/1000 PD for the injectable form and 137.82 DDD/1000 PD for the inhaled form. In contrast, only 11 patients received fosfomycin, with a consumption of 12.61 DDD/1000 PD. Colistin prescriptions were microbiologically documented in 63.63% of cases, whereas all fosfomycin prescriptions were documented. The main pathogens responsible for infections treated with colistin were *Pseudomonas* spp. (38.9%) and *Acinetobacter baumannii* (32.73%). For fosfomycin, the most frequently isolated pathogens were *Pseudomonas aeruginosa* (45.45%), *Proteus mirabilis* (27.27%), and *Providencia* spp. (18.18%). Although both antibiotics belong to the ‘Reserve’ group of the WHO AWaRe classification, fosfomycin dispensing was more restrictive due to its higher cost and the requirement for a nominative request accompanied by an antibiogram from the pharmacy department.

**Conclusions:**

Last-resort antibiotics are widely prescribed for the management of critically ill patients in burn intensive care units. However, their use should be strictly monitored and continuously evaluated in order to limit the emergence of antimicrobial resistance.

